# The maternal social environment shapes offspring growth, physiology, and behavioural phenotype in guinea pigs

**DOI:** 10.1186/1742-9994-12-S1-S13

**Published:** 2015-08-24

**Authors:** Nikolaus von Engelhardt, Gabriele J Kowalski, Anja Guenther

**Affiliations:** 1 Animal Behaviour, Bielefeld University, Morgenbreede 45, 33615 Bielefeld, Germany

**Keywords:** maternal effects, social, density, prenatal, development, sex-specific, growth, behaviour, guinea pig, Cavia aperea f. porcellus

## Abstract

**Background:**

Prenatal conditions influence offspring development in many species. In mammals, the effects of social density have traditionally been considered a detrimental form of maternal stress. Now their potential adaptive significance is receiving greater attention.Sex-specific effects of maternal social instability on offspring in guinea pigs *(Cavia aperea f. porcellus) *have been interpreted as adaptations to high social densities, while the effects of low social density are unknown. Hence, we compared morphological, behavioural and physiological development between offspring born to mothers housed either individually or in groups during the second half of pregnancy.

**Results:**

Females housed individually and females housed in groups gave birth to litters of similar size and sex-ratios, and there were no differences in birth weight. Sons of individually-housed mothers grew faster than their sisters, whereas daughters ofgroup-housed females grew faster than their brothers, primarily due to an effect on growth of daughters. There were few effects on offspring behaviour. Baseline cortisol levels in saliva of pups on day 1 and day 7 were not affected, but we saw a blunted cortisol response to social separation on day 7 in sons of individually-housed females and daughters of group-housed females. The effects were consistent across two replicate experiments.

**Conclusions:**

The observed effects only partially support the adaptive hypothesis. Increased growth of daughters may be adaptive under high densities due to increasedfemale competition, but it is unclear why growth of sons is not increased under low social densities when males face less competition from older, dominant males. The differences in growth may be causally linked to sex-specific effects on cortisol response, although individual cortisol response and growth were not correlated, and various other mechanisms are possible. The observed sex-specific effects on early development are intriguing, yet the potential adaptive benefits and physiological mechanisms require further study.

## Introduction

In many species, social density during reproduction affects maternal investment. In pregnant or lactating females, the social environment can influence maternal hormonal or nutritional state, which in turn can alter the transfer of hormones or resources to offspring, thereby modifying offspring growth, physiology and behavioural development [1,2]. Such maternal influences on offspring phenotype have traditionally been considered detrimental consequences of maternal stress [3,4]. More recently, however, increased attention has been given to the idea that they may represent adaptive maternal effects which prepare offspring to deal with the challenges of the environment into which they will be born [1,5,6]. It has been proposed that high maternal social densities predict a less stable and more competitive environment for offspring, whereas low social densities forecast a less competitive environment and higher predictability of social encounters for offspring [2,7].

An extensive body of studies have investigated the effects of prenatal social conditionssuch as crowding, frequent exchange of group members or placing animals in unfamiliar social environments [8-13]. Often these conditions led to lower offspring birth weight, followed by reduced growth rates in one or both sexes [14-16]. However, not all studies investigating the effects of social density or maternal stress found reduced growth. Increased growth was found in offspring of prenatally stressed, captive rats *(Rattus norvegicus)*[17], ewes (*Ovis aries*)[18], and cows *(Bos taurus)*[19] .In free-living red squirrels (*Tamia sciurus hudsonicus) *increased growth under simulated high densities appears to be adaptive [20]. Finally, effects of the maternal social environment or maternal stress are frequently sex-specific. These species-specific, context-dependent or sex-related effects of prenatal conditions on offspring growth further support the idea that they are not detrimental consequences of maternal stress but represent adaptations to variation in ecological or social conditions.

In addition, the maternal social environment was frequently found to affect offspring behaviour and physiology. Crowding of pregnant mice *(Mus musculus)*, for example, led to higher amounts of social interaction in offspring, less activity and increased defecation in unfamiliar environments [13]. Pups of female rats who experienced social instability during pregnancy showed more anxiety-related behaviour and reduced activity later in life [21].

Surprisingly, in contrast to high social densities, the effects of low social densities during pregnancy have hardly been investigated. Offspring of individually housed female mice showed reduced growth [22], and sows (*Sus scrofa*) housed individually during pregnancy gave birth to offspring that were lighter, drank more, and vocalized more when isolated [23]. These effects resemble those found under high densities or maternal stress and have also been interpreted as detrimental effects of maternal stress on offspring condition and ability to cope with stress.

In the present study, we studied the effects of individual housing in guinea pigs (*Cavia aperea f. porcellus*) during the second half of pregnancy, representing the most extreme case of low social density, in comparison with social housing in small mixed-sex groups, a more normal social situation. We focused on offspring development during the first five weeks after birth, a period which has been studied in detail only in very few species. We would expect effects to be most pronounced and relevant during this phase of life, particularly in a highly precocial species like the guinea pig. Guinea pigs are a very social species in which males defend small harems of between 1 and 7 females [24]. As has been shown in other rodents [25-27], population densities can fluctuate over relatively short time scales, also in wild cavies, the wild congeners of the guinea pig [28-30]. Social density can therefore differ strongly between populations and years resulting in predictable differences in optimal life history and reproductive strategies within and between generations that may be anticipated by maternal effects on offspring development. In guinea pigs, unstable social conditions during pregnancy are thought to be associated with high social densities under natural conditions, and have been found to cause behavioural infantilisation of male offspring after weaning in captivity [12]. In female offspring, the same prenatal treatment led to behavioural and physiological masculinisation [11]. These characteristics have been interpreted as adaptations to high population densities [7], since masculinised females may be better at defending limited resources, whereas young males may benefit from avoiding agonistic encounters with dominant males. Female guinea pigs housed in unstable social conditions also produced female-biased offspring sex ratios [31]. Effects of the maternal social environment on immediate postnatal growth, behaviour and physiology have not been investigated in guinea pigs, to our knowledge.

Our study, conducted in two independent replicates (batch 1 and batch 2, see methods), focused on the effects of maternal social housing conditions on offspring growth, behaviour and stress physiology. Based on the idea that maternal effects on offspring represent adaptations to social density, we expected individual housing to have opposite effects to those typically found under social instability or high density situations. We therefore expected that individual housing should positively affect growth, reduce stress responsiveness and anxiety-related behaviour and/or increase boldness and exploration behaviour. Given the sex-specific effects of social instability in guinea pigs, we also expected that low density might especially have positive effects on sons who do not have to avoid dominant males under these conditions. Alternatively, individual housing may be perceived by mothers as predictive of adverse conditions, resulting in reduced growth, increased anxiety and stress responsiveness in one or both sexes.

## Results

### Litter size, sex ratio and pup growth

Litter size, ranging between 1-5 pups (group housing: 3.2 ± 0.3, individual housing: 3.3 ± 0.4; all results are shown as mean ± SEM), was not significantly influenced by the maternal treatment (t=0.38, p=0.7). Sex ratios at birth also did not differ between treatments (z=-0.59; p=0.56, n=84). Sex ratios were not related to litter size (z=0.85, p=0.4)

**Fig. 1:**
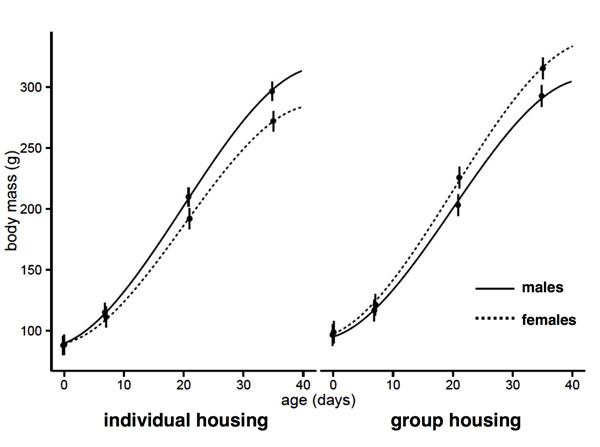
Offspring growth. Body mass (means ± SEM estimated separately for each age, with fitted lines from the mixed model) of female (dotted lines, individual: n=18, group: n=17) and male (continuous lines, individual: n=25, group: n=19) offspring of individually housed mothers (left) and group-housed mothers (right). Daughters of individually-housed females had reduced growth and daughters of group-housed females had increased growth (significant three-way interaction between the maternal treatment, offspring sex and age (F_1,245_=16.15; p=0.0001)

Birth weight (group housing: 97.9 ± 4.7 g, individual housing: 93.2 ± 5.2 g) and body length at birth (group housing: 12.6 ± 0.36 cm, individual housing: 12.5 ± 0.4 cm) did not differ significantly between pups of the maternal treatments or between sexes (all p>0.07). Although all pups started from approximately the same mean birth weight, the treatment had opposite effects on growth of female and male pups (significant three-way interaction between the maternal treatment, offspring sex and age (F_1,245_=16.15; p=0.0001, figure 1). This effect remained significant when including litter size in the model, which had a negative effect on growth (F_1,22_=12.21; p=0.002). The offspring of batch II were lighter on average than those of batch 1 (F_1,25_=23.7; p=0.0001), but the three-way interaction was found in both batches when analysed separately (batch I: F _1,101_=5.5; p=0.02, batch I: F _1,124_=4.2; p=0.04). When analysed separately by sex, daughters of individually-housed females grew significantly slower than daughters of group-housed females (interaction between treatment and age: F_1,104_=15.7; p=0.0001), but the positive effect on growth of sons was not significant (interaction between treatment and age: F_1,130_=2.0; p=0.15). A separate analysis for each treatment showed that daughters of individually housed females grew significantly slower than their brothers (interaction between sex and age: F_1,137_=16.5; p=0.0001), while the opposite was found for offspring of the group-housed mothers (interaction between sex and age: F _1,106_=4; p=0.048). There were similar trends for differences in offspring length, but the effects did not reach significance (three-way interaction between the maternal treatment, offspring sex and age (F_1,238_=1.5; p=0.2; data not shown).

### Behaviour

To evaluate potential treatment effects on offspring behaviour, we used a number of tests to assess different behavioural categories (see methods). Our expectation was that the *Struggle test*, *hand-escape test* and *novel environment test*would measure anxiety or emotionality. The *novel object test* was used to assess boldness and the *social separation test* to measure sociability. Correlations between tests indicated that indeed the latency to leave hand and the struggle duration were correlated, thus representing different measures of the same behavioural category (see table 1). Duration of activity in a novel environment, however, was not correlated to either of the variables. Instead, it correlated positively with the number of calls emitted during a brief social separation.

**Table 1:** Correlations between behavioural traits.

age	trait	Log (duration of struggling)	Log (latency to leave the hand)	activity	number of calls	Log (latency to reach the mother)	duration of activity
day of birth	log (duration of struggling)	–					
	log (latency to leave the hand)	**-0.4 (<0.01)**	–				
day 1	activity	0.06 (n.s.)	-0.14 (n.s.)	–			
	number of calls	0.07 (n.s.)	-0.16 (n.s.)	0.05 (n.s.)	–		
	log (latency to reach the mother)	-0.18 (n.s.)	0.23 (n.s.)	**-0.43 (<0.01)**	-0.04 (n.s.)	–	
day 8	duration of activity	-0.09 (n.s.)	-0.02 (n.s.)	0.17 (n.s.)	**0.31 (<0.05)**	-0.06 (n.s.)	–

Correlations between the different behaviours calculated as Pearson's correlation coefficients with significance levels in brackets up to a level of p=0.08. Non-significant correlations are indicated by (n.s.).

**Fig. 2:**
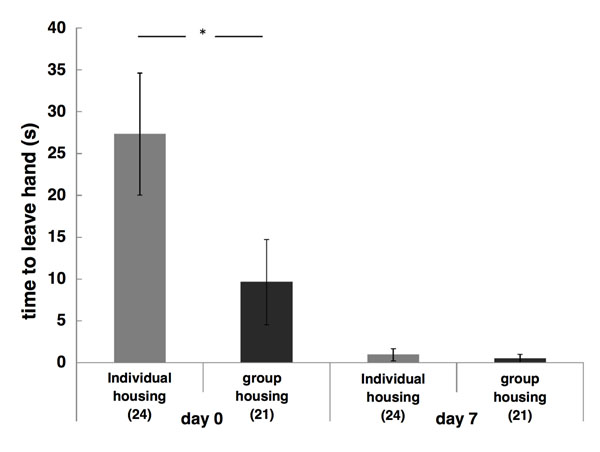
Hand-escape latency. Mean latency (± SEM) to leave the hand of newborn pups (day 0, left) and on day 7 (right). Newborn pups from individually housed mothers needed significantly more time to leave the hand than pups from group-housed mothers (F_1,12_=5.38; p=0.03). This difference disappeared within the first week.

On the day of birth (measured only in batch II), pups from individually housed mothers had a longer latency to leave the hand compared to pups from group-housed mothers (F_1,12_=5.38; p=0.03, figure 2). One week later, all pups left the hand almost immediately and there was no longer a significant difference between treatments (F _1,12_=0.87, p=0.37).

Struggle duration, activity, number of calls, and latency to reach mother during the social separation test did not differ significantly between pups from the different maternal treatments (all F-values < 3.4, all p > 0.1).

Struggle duration (F_1,83_=242; p=0.0001), activity (F_1,37_=16; p=0.0001), number of calls (F_1,37_=8.7; p=0.006) and latency to reach the mother (F_1,37_=12.1; p=0.001) during the social separation test were significantly influenced by the age of the pups (measured at two ages only in batch I). All pups struggled more, became more active and were faster to reach their mother over time and emitted fewer calls.

**Fig. 3:**
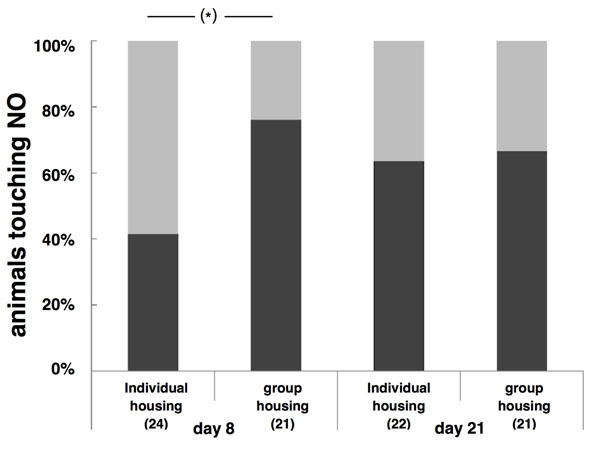
Novel object test. Number of offspring (in percent) interacting with a novel object on day 8 and day 21. On day 8 there was a trend (p=0.059) for more pups of group-housed mothers to touch a novel object than pups of individually housed mothers. On day 21 there was no difference. This test was only conducted with the second batch, see table 2.

In the novel environment test conducted on day 8 (only batch II), the duration of exploration activity in the first 2 minutes after a pup was introduced to the novel environment did not differ significantly between treatments (individual housing: 25.0 ± 3.2, group housing: 26.2 ± 6.4, F_1,12_=0.03, p=0.9). However, there was a trend towards more pups from the group treatment approaching the hut (boldness; z=-1.89; p=0.059, n=45, figure 3). When repeating the novel environment test on day 21, there was again no significant difference between the treatments in duration of exploration activity (individual housing: 19.2 ±1.9, group housing: 22.1 ±4.1; F_1,11_=0.4, p=0.6) nor a difference in the probability to approach the hut (z=0.23, p=0.8, n=43, figure 3). We found no significant effect of the interaction between treatment and sex nor significant sex differences in any behavioural test (all p > 0.10).

**Table 2:** Measurement schedule.

date	test	measurements	batch	*n_individual_/n_group_*
day of birth		weight, length	I/II	47/37
	struggle	duration of struggling	I/II	47/37
	hand-escape	latency to leave the hand	II	24/21
day 1	cortisol	cortisol baseline activity	I/II	43/33
	social separation	activity, number of calls, latency to reach the mother	I/II	47/37
day 7		weight, length	I/II	44/37
	struggle	duration of struggling	I/II	44/37
	social separation	activity, number of calls, latency to reach the mother	I	20/16
	hand-escape	latency to leave the hand	II	24/21
day 8	cortisol	cortisol baseline	I/II	41/35
		cortisol response to social separation	I/II	39/34
	novel environment	duration of activity	II	24/21
	novel object	approach to novel object (yes/no)	II	24/21
day 21		weight, length	I/II	44/37
	novel environment	duration of activity	II	22/21
	novel object	approach to a novel object (yes/no)	II	22/21
day 35		weight, length	I/II	44/36

Overview of the tests conducted, the order and the variables measured. Additionally, the column “batch” indicates whether a specific test was conducted in both batches or only in one of them. The last column gives the sample sizes separated for maternal treatment.

### Cortisol

**Fig. 4:**
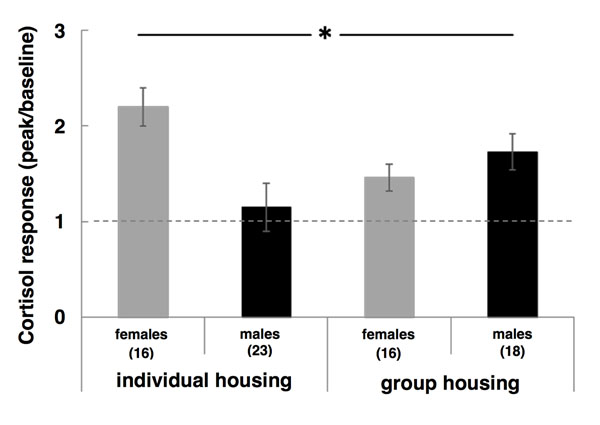
Cortisol response. Cortisol response in saliva on day 8(ratio of cortisol concentration in saliva after 30 min social separation over baseline cortisol concentration) was reduced in sons of individually-housed females and daughters of group-housed females (significant interaction between treatment and sex, F_1,44_=5.96; p=0.02).

Baseline cortisol of pups did not differ between treatments the day after birth (F_1,23_=0.01; p=0.9) or between sexes (F _1,51_=3.2; p=0.15). One week later, baseline levels still did not differ between treatments (F_1,22_=0.04; p=0.85) or between sexes (F_1,49_=2.1; p=0.16). At one week of age, we also tested cortisol response to a brief (30 min) social separation. We found a significant interaction between treatment and sex (F_1,44_=5.96; p=0.02, see figure 4). Cortisol response on day 7 was highest in daughters of individually-housed females and sons of group-housed females and lowest in sons of individually-housed females and daughters of group-housed females. When tested separately for each offspring sex or treatment, there were no significant effects of treatment or offspring sex, respectively (all p>0.1). Cortisol response was lower in batch II than in batch I (F _1,22_=19.2; p<0.001), but the effect of the interaction between treatment and sex was found in both batches when analysed separately: batch I: F _1,32_=4.6; p=0.04, batch II: F _1,35_=4.1; p<0.05. This sex-specific effect suggests a possible link to the sex-specific effect on offspring growth, although cortisol response did not significantly predict offspring growth when included as a covariate in the model testing the effects of treatment, sex and age on offspring weights (F_1,22_=1.49; p=0.23). Baseline cortisol levels were also affected by batch: in batch II, baseline cortisol levels on day 1 (F_1,22_=21.4; p<0.001) and day 7 (F _1,22_=30.7; p<0.001) were higher than in batch I.

## Discussion

In many species, the maternal environment has strong effects on offspring development, which has mostly been studied with a focus on the detrimental effects of maternal stress, especially in mammals. Only recently have potential adaptive explanations been considered, resulting in a broader perspective of when mothers may adaptively adjust offspring development to conditions anticipated by the mother due to her experience[1,2,5]. We tested the effects on pups’ development of individual housing compared to group housing of mothers during pregnancy. We expected individual housing to have opposite effects to those in studies simulating high social densitiesby crowding or unstable social condition. Our results overall show a strong, sex-specific modulation of offspring growth and some indications for effects on physiology and behaviour, even this early in life. Under individual housing, daughters grew more slowly than sons, whereas the reverse was observed under group housing. In addition, daughters of individually-housed mothers showed the highest cortisol response to social separation, while sons showed the lowest response. These were robust effects observed in two replicate experiments despite differences in growth and hormone levels between the two batches. Further studies with different group sizes and in wild cavies are needed to investigate how the effects of the maternal environment are transmitted from mother to offspring as well as how the effects of low social densities differ from effects of high densities, and whether they represent adaptations to the social environment.

### Offspring growth

In previous studies in guinea pigs, different maternal treatments also led to sex-specific effects on offspring growth. Non-social maternal stress generally seemed to have positive effects on growth of daughters and/or negative effects on growth of sons [32-34]. However, maternal social instability in guinea pigs, which is thought to simulate high social density, did not affect offspring growth, although males and females were never compared directly [11,35,36]. We observed increased growth in daughters of group-housed females and reduced growth in daughters of individually housed females and no effect on male growth. Increased growth in daughters of group-housed females may support the adaptive hypothesis since it has been suggested that females may benefit from increased competitiveness under higher densities [7]. We did not find the opposite positive effects on male growth under low densities, which would also be expected since males may benefit from rapid growth under low social densities when they stand a higher chance of gaining a dominant position and reproducing early in life. From an adaptive perspective, long-lasting effects on survival and reproduction are especially relevant. In a recently published follow-up study of this experiment, we showed that sons of individually housed mothers have higher reproductive success compared to sons of group-housed mothers under low social densities [37], suggesting that males indeed benefit when their mothers are housed individually during pregnancy.

### Behaviour

We investigated the effects of the maternal social environment on behaviour in a variety of tests thought to reflect different underlying behavioural categories (for details see methods). In this study, only hand-escape latency, a measure of anxiety, was significantly influenced by the maternal treatment: Offspring of individually housed females needed longer to leave the hand of the observer but only on the first day of life. Other anxiety-related behavioural traits were not influenced by treatment. In the novel object test, which was used to assess boldness, there was a tendency for fewer offspring of individually housed females to touch a novel object in the novel environment task on day eight. Thus, offspring of individually housed females may be regarded as more shy. However, by testing on day 21, treatment differences had disappeared, suggesting no strong effect on boldness. Sociability, assessed by measuring activity, number of calls and time to reinstate contact with the mother in the social separation test, was not influenced by the maternal treatment. The weak effect on anxiety and boldness in offspring of individually-housed mothers is contrary to our expectation that individual housing has opposite effects to high social densities. Instead it supports the idea that individual housing is perceived as a stressful, adverse environment, since adverse maternal conditions such as crowding, isolation and non-social maternal stress seem to result most commonly in an increase in anxiety-related behaviours, suppression of activity or delay in behavioural development [13,38-41]. However, some studies also find the opposite outcome [41], hence more data is clearly needed. Also, we performed multiple behavioural tests at two stages in life and found only very few significant effects, therefore it is possible that the observed trends are spurious effects.

The general lack of treatment differences in most of the observed behavioural traits was surprising since earlier studies in guinea pigs found clear effects of the maternal environment on behaviour, including activity and emotionality traits [11,12,33,42,43]. Strong behavioural and physiological differences very early in development were also found in relation to the maternal photoperiod and sibling-size rank in the cavy, the presumed wild congener of the domesticated guinea pig [44-46]. We did find the same negative relationship between the duration of struggling and the latency to leave the hand that was found in wild cavies [46], which suggests that the tests reliably reflect underlying behavioural traits. However, comparative studies on wild and domesticated guinea pigs suggest that emotionality may not be a very stable trait in the domesticated guinea pig [47,48], possibly due to domestication effects. We may therefore have measured behaviours that are not strongly affected by the maternal environment. Also, previous studies usually focused on older offspring that had reached independence [11,12,33,42,43], whereas our measures were taken earlier in life when effects may be less pronounced. Effects may thus be observed only later in life, for example because they are mediated by postnatal maternal behaviour as suggested by other studies in rodents [22,49-51] (see also below). Finally, as highly precocial animals, guinea pigs are born at a very advanced stage of development compared to most other studied species which are usually altricial. Developmental mode may therefore also partly explain why we find no or only very weak effects on offspring behaviour. Further studies on the effects of the maternal social environment on early postnatal behaviour of offspring and mothers, especially in species differing in developmental mode are thus clearly needed.

### Cortisol levels

In our study, baseline cortisol levels in saliva were not affected by the treatment. Cortisol response to social separation was blunted in sons of individually-housed mothers and daughters of group-housed mothers. In contrast, daughters of individually-housed mothers and sons of group-housed mothers, showed a clear, approximately two-fold, increase of salivary cortisol levels after 30 minutes of social separation. This resembled a similar increase found earlier in plasma in response to social isolation [52] or in saliva as a consequence of a non-social, postnatal [53] or maternal stressor [43]. Previous studies on the effects of the maternal social environment did not measure cortisol response and also found no effect of social instability on baseline cortisol levels in female offspring, although adrenal weights were increased [11]. Male offspring, on the other hand, had a delayed maturation of the HPA axis as shown by a later decrease of baseline cortisol during development [12]. Cortisol response was increased in sons and decreased in daughters of prenatally-stressed females in the above-mentioned experiments which also resulted in sex-differences in offspring growth [33,43]. Since these effects on cortisol response are again opposite to what we find in offspring of individually-housed females, social stimulation in group-housed females may indeed have similar effects as maternal stress, perhaps by changing general activity levels and metabolism.

### Mechanisms

How do environmental factors act on mothers? How are the effects transmitted to offspring? And how is offspring development modified? Since all females experienced a change in the number of males and females in their group, we cannot know whether the absence of males, females or both is responsible for the effects observed. The perception of the social environment may directly affect females, for example by changing the maternal nutritional and energetic environment. Prenatal conditions can also modify maternal endocrine status which can influence offspring morphology, physiology and behaviour since maternal hormones, such as adrenal corticosteroids and androgens or gonadal estrogens and progestins, can cross the placenta [54,55]. Sex-specific maternal effects may be due to sex-specific susceptibility of offspring or differential maternal allocation of resources or hormones to male and female offspring [56,57]. Adrenal corticosteroids and androgens are frequently studied candidates which change in relation to social and nutritional challenges. They can be transmitted to offspring across the placenta and affect early growth and gonadal and brain organisation [7,58,59]. In guinea pigs, the significance of maternal cortisol levels is somewhat unclear since they increase to very high levels during pregnancy, but this may not translate to significant elevation of embryonic exposure to cortisol [7]. Alternatively or in addition, elevated cortisol levels in the maternal plasma seem to reduce androgen levels so that effects on offspring may also be caused by altered transfer of androgens to the developing embryo [7]. As mentioned earlier, differences in the maternal environment during pregnancy may also cause differences in maternal behaviour towards offspring after birth. Prenatally stressed female guinea pigs showed higher postnatal aggression towards their offspring [60]. If individual housing influenced maternal behaviour in our study, this might have changed their interaction with their offspring and affected their development. Unfortunately maternal behaviour was not studied in our experiment.

## Conclusions

Variation in the social environment is highly important for reproduction and survival in many species and can strongly affect offspring development. So far, maternally transmitted effects of the social environment have mostly been studied with a focus on the detrimental consequences of prenatal stress. More attention should be given to possible adaptive maternal effects on offspring in response to variation in the social environment. We find strong sex-specific effects on offspring growth and cortisol responsiveness that are consistent with an increased investment in daughters when social density is high. Increased investment in daughters may be beneficial for group-housed mothers since female competition is increased under high social densities compared to low social densities. Sons do not benefit from increased maternal investment since they cannot out-compete dominant adult males under these conditions. However, we do not find the predicted increased investment in sons when social density is low.

The observed effects may be mediated by variation in social stimulation when housed in different social groups which may have similar effects to other environmental stimuli during pregnancy, including factors generally considered as stressors. Whether these effects are transmitted to offspring through hormonal programming, maternal resource transfer or postnatal maternal behaviour requires additional studies. Due to a lack of data on early development under different pre- and postnatal social conditions in guinea pigs and other species, it is currently unclear whether these should be interpreted as detrimental effects of maternal stress or beneficial effects on offspring development in anticipation of the postnatal social environment. Further studies on the functional consequences in different pre- and postnatal social settings and in wild cavies in their natural environment will be essential to understand the extent to which observed effects represent detrimental consequences of maternal social stress or adaptive shaping of individual phenotypes.

## Methods

### Ethical note

The experimental procedures were conducted in accordance with German animal protection laws. Animal facilities were approved (dated 18 April 2002) for keeping and breeding guinea pigs for research purposes by the local government authority responsible for health, veterinary and food monitoring (Gesundheits-, Veterinär- und Lebensmittelüberwachungsamt) under the licence number 530.42 16 30-1.

### Subjects and housing conditions

The experiment was conducted in two batches, using descendants of outbred, multicoloured and shorthaired breeding stocks from the Universities of Bielefeld (batch I) and Münster (batch II). Due to frequent exchange of animals, the breeding stocks of both universities are rather similar genetically. Animals in batch II were moved shortly after independence from the University of Münster to Bielefeld. All adult animals and their offspring could clearly be individually distinguished by their natural fur colours and patterns which were recorded by taking pictures on the day of birth.

Animals were housed under natural light conditions with additional artificial light from 6 a.m. to 8 p.m. and a temperature of 20 ± 3°C. Wood chips were used for bedding, several plastic huts provided cover, and food and water was available at several feeding stations. Pellet food (guinea pig chow, Höveler, Langenfeld, Germany), hay, and water were provided ad libitum, and lettuce, carrots or bell pepper were given every other day. Additionally, drinking water was supplemented with vitamin C (ascorbic acid, approximately 1 g/l) once a week.

All experimental animals were housed shortly after weaning in a single large group (one group for each batch) comprising fourteen immature females and two immature males in a 15 m^2^ enclosure. At the time the groups were founded (spring 2011 and autumn 2011), neither females nor males had reached sexual maturity. Two months later, females were randomly assigned to one of the two different treatments (individual housing vs. group housing). In both batches, eight females were assigned to individual housing conditions while six females were assigned to group housing conditions. These females stayed together with the remaining females of their original group, resulting in one group of six females for each batch. All females were pregnant at this time. All animals were moved to new enclosures in a different room on the day the treatment groups were formed to ensure both group and individually housed females experienced a change of cages and rooms. Individually housed females were moved into standardised 0.8 m^2^ enclosures with wood chips for bedding, one hut for cover, a feeding dispenser and a water bottle. Females assigned to the group treatment remained together with the other females assigned to the group treatment but were moved into a new 8.5 m^2^ enclosure in the same room as the individually housed females, with several huts, feeding dispensers and water bottles. All animals had acoustic and olfactory contact, but individually housed females were prevented from having visual or social interaction with other animals. The six group females stayed together with the males until shortly before parturition. Two days before the estimated date of parturition (mean age of maturity + mean duration of pregnancy of 68 days), the two males were removed from the group-housed females to prevent post-partum pregnancies.

Each enclosure was subsequently checked daily in the morning and in the late afternoon for newborn pups. All females gave birth successfully, although one mother died shortly after parturition. Birth dates, litter size, mass and sexes of pups were noted within 12 hours after birth. In total, 91 pups were born: 43 in the first batch and 47 in the second batch. Six pups from two females were excluded from the experiment because they only conceived shortly before the treatment started and therefore differed strongly from the other females. Individually housed females were, on average, pregnant for 21.5 ± 4.5 days and group-housed females for 22.1 ± 5 days when placed into the treatments. There was no difference in pregnancy stage between treatments (F_1,21_=0.12; p=0.74) or between batches (F_1,21_=0.8; p=0.36). One son of a group-housed female was stillborn and only included in the analysis of birth sex-ratios, resulting in a final sample size of 84 offspring for the other measures on the day of birth. Four more offspring died before the end of the experiment (a daughter and two sons of two individually-housed females and a daughter of a group-housed female) and were included in the experiment until the day they died. These unexplained deaths were unlikely to be related to the treatments and the exclusion of these pups from the analysis slightly changed some estimates but not the overall significance of the effects.

**Fig. 5:**
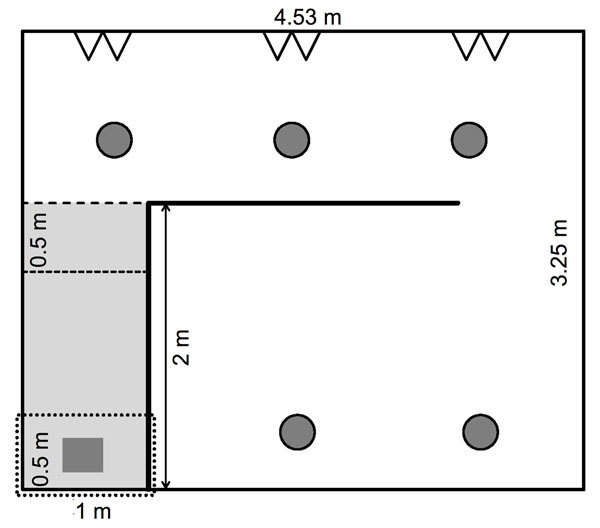
Enclosure with test arena. Offspring and their mothers were housed in a single group in this enclosure. Gray circles indicate feeding stations, open triangles indicate water bottles. The hand-escape test was conducted in a smaller compartment of the main enclosure indicated by the dotted line in the lower left corner.For the social separation test, a 2 x 1 m^2^ compartment was separated from the main enclosure by non-transparent walls. From this test arena, a smaller compartment (1 x 0.5 m^2^) was subdivided by wire mesh. In the larger compartment of this test arena, we placed a small cage made of wire mesh under which the mother was placed beneath a hut. The pups were caught one after the other and placed into the smaller compartment. They could thus see and hear their mother but could not reach her.

After a female had given birth, the mother and her young were transferred into a 15 m^2^ enclosure with other mothers and their pups (see figure 5). Prior to this transfer, we placed one mother and her two pups not belonging to the experiment in the enclosure so that the first experimental mother would not be alone. For the consecutive mothers, the group consisted of all the other mothers and pups present in the enclosure at the time of transfer. The mean time from separation of males until parturition was 12 days, ranging between 2 and 18 days, and females gave birth on average 45.9 ± 4 days after they were assigned to the housing conditions.

There were slight differences in the initial formation of the groups between the two batches. In batch I, the fourteen nulliparous females and two males were housed together when all animals were approximately 30 days old, ranging between 25 and 35 days of age. The females were derived from eleven litters, the males from two additional litters. Female guinea pigs born in spring mature at around 45 days, as do their wild counterparts [61]. Male guinea pigs mature at between 50 and 70 days of age [62]. Thus, the group was given approximately one month to form stable bonds between individuals before animals started reproducing. In batch II, all females used for breeding came from different litters and the fourteen females were put together shortly after weaning, as in the first batch. The two males were added to the females two weeks later than in batch I because we first genotyped all individuals at 12 variable microsatellite loci [30,63] to select the males least related to each other and the females. Both female and male guinea pigs mature later in autumn than in spring [61]. Consequently, the time the group remained together before onset of reproduction was approximately the same as in the first batch, as confirmed by the fact that the stage of pregnancy did not differ between treatments (see above). Otherwise, the experimental procedures, including housing conditions, were the same as in the first batch.

### Behaviour

We assessed anxiety/emotionality of all pups born during the experiment using a *struggle test, hand-escape test* and *novel environment test *[46] (for details of the tests see below). We assessed boldness by means of a *novel object test* and sociability through a *social separation test*. Struggle duration, hand-escape latency and exploration behaviour in an open field test were correlated in the wild congener of the domesticate guinea pig, the wild cavy, indicating that all of these behaviours may reflect the same underlying behavioural category [46]. Hand-escape latency and open field behaviour were also correlated to cortisol baseline levels, hence potentially representing a measure of anxiety [45,46]. Furthermore, struggle duration and hand escape latency showed temporal consistency even in two to three-day-old juveniles, suggesting that these tests are suited to measure behavioural phenotype in early life-stages. Approaching a novel object is often regarded as boldness or curiosity [64] and represents a different behavioural category than anxiety or emotionality in guinea pigs [65]. To measure how pups of the different treatments react to a brief social separation from their mother, we adopted a well established separation paradigm, in which guinea pigs of all ages react strongly with behavioural, physiological and immunological changes [66]. To test whether the behaviours that we measured represent different behavioural categories or the same underlying trait, we correlated the variables of each test with each other (see table 1). We correlated only the first measurements of each test with each other to exclude any possible influences of habituation or carry-over effects between repeated test situations. Most variables were not correlated with each other even when measured during the same test (e.g. social separation test). In cases where we found significant correlations (3 cases), the strength of correlation was only moderate (ranging between 0.3 and 0.43), so that we decided to analyse each behaviour separately rather than computing composite variables.

### Measurement schedule

(see table 2 for overview)

#### Day of birth

Body mass, length and behaviour of all pups in a litter were measured within the first twelve hours after birth. If they were still wet when found, the first measurements were taken at the subsequent check when they were completely dry and able to walk, indicating that at least two hours had passed since birth. As mothers were accustomed to the observers, they did not flee or show signs of distress, and pups could be removed calmly from their side.

##### Struggle test

Each pup was removed singly from its mother, gently turned and held on its back in the hand of the observer and the total duration the animal's struggle during thirty seconds was recorded by starting and stopping a stopwatch each time the animal started and stopped moving (duration of struggling). Afterwards, each pup was weighed and its length (from snout to tail bone) was recorded. After the measurements, the pup was placed in a dark transport box. After all juveniles had been measured, the mother was also put into the transport box, and the family was transported to the room and enclosure that contained all other pups and their mothers.

##### Hand-escape test

In the second batch, an additional behavioural test was conducted immediately before the family was released into the new enclosure. For this test, the mother was placed beneath a hut in a small compartment (1 x 0.5 m^2^, see figure 5) separated from the main enclosure by an opaque plastic wall. The pups were then removed one by one from the transport box and placed into the compartment on the observer's outstretched hand. From this vantage point they could see their mother at a distance of approximately 60 cm. We then measured the time they took to leave the hand (latency to leave the hand). If the pup did not leave the hand within sixty seconds, it received the maximum score and was placed beside its mother. The dividing wall was removed after all juveniles were released so that the whole family could move into the main enclosure.

#### Day after birth

Between 9:30 and 11:00 in the morning, each pup was caught individually and two saliva samples were taken for later cortisol analysis using a Q-tip (cotton-swab). Sampling started less than a minute after disturbing animals for capture. During the sampling, the pup was placed on the lap of the observer. Both sides of the Q-tip were used to collect two consecutive samples within a few minutes that were analysed separately and then averaged. The Q-tip was cut in the middle and placed with the cotton part downwards into a 0.5 ml eppendorf tub that had been pierced at the bottom. The pierced eppendorf tube containing the Q-tip was placed into a 2.0 ml eppendorf tube and stored on ice for up to 1 hour before further processing (see below).

##### Social separation test

This test was performed one day after birth in the afternoon (between 2 pm and 4 pm). For testing, a smaller compartment was temporarily separated off within the main enclosure (see figure 5), and pups were caught one after the other for testing. As soon as a pup was placed in the test arena, we measured the cumulative duration of activity for thirty seconds (activity) with a stop watch. We scored as activity each movement that resulted in a change of body position (excluding head movements). Afterwards, we counted the number of calls the pup emitted within thirty seconds (number of calls). After that, we removed the wire mesh wall and measured the time it took the pup to reach its mother (latency to reach the mother). One pup made no attempt to reach its mother within three minutes, so the test was stopped and the maximum latency of 180 s was used for analysis.

#### One-week-old pups

Each juvenile was caught singly in the morning (9:30 a.m. to 11:00 a.m.) when it was seven days old. First the struggle test was repeated, then body mass and body length were measured. In batch II the hand test was also repeated. For practical reasons the social separation test was conducted again on the afternoon of day seven only in batch I.

##### Novel environment test and novel object test

Instead of the social separation test, in batch II we measured exploration (duration of activity) of a novel environment and boldness (approach to a novel object) on day eight. The novel environment test lasted for two minutes and was conducted in the same room where the animals were housed. The test animal was removed from the group and placed into a 0.5 m^2^ box with fresh wood chips covering the floor. The duration of activity within the first two minutes was measured, scoring each movement that resulted in a change of body position (excluding head movements). After two minutes, the animal was randomly placed in one of the corners of the box and a novel hut with a different shape and colour than the huts the animals were familiar with was placed in the opposite corner of the box. As a measure of boldness, we recorded how long it took the animals to touch that novel object using a maximum latency of two minutes. Unexpectedly, a large proportion of animals did not touch the novel object within the two minutes. We therefore assigned a score of 1 or 0 for the analysis, depending on whether or not the animal touched the novel object within the two minutes.

During the morning of day 8, each pup was again caught singly and another saliva sample was taken as described above. After the initial sample, the pup was introduced into a 30 x 40 cm plastic box from where it could hear and smell other animals but not see them. It was left there for 30 minutes before we took a second saliva sample to measure cortisol response to social separation.

#### Weaned juveniles (day 21 and day 35)

When pups were 21 days old (around weaning) and 35 days old (independence), body mass and body length were measured (between 9:30 a.m. and 11:00 a.m.). In the second batch the novel environment test was conducted again on day 21, except for two animals that were overlooked.

One of three trained observers took measurements and made direct observations of behaviour according to a pre-planned schedule that was unbiased with respect to the experimental treatment and observer. To ensure uniform recording of behaviour, we used unequivocal measures established during previous experiments. Observers were always visible to the animals during testing. Observers knew which pups belonged to which experimental treatment on the day of birth when they removed pups from their individually housed or group-housed mothers. Afterwards, all pups were housed in one large group and observers were not aware of the experimental treatment of individuals during testing.

#### Cortisol measurements

All samples on a given day were immediately stored on ice for up to 1 hour. They were then centrifuged for 10 minutes at 5000 rpm to spin the saliva from the cotton through the pierced small eppendorf tube into the larger eppendorf tube. All samples were frozen at -20° Celsius until further analysis by enzyme immunoassay, following the instructions of the manufacturer (Demeditec DES 6611, sensitivity 14 pg/ml and cross-reactivity with all tested endogenous steroids < 2%). For the assay, thawed saliva samples were centrifuged again and 5-20 µl samples were diluted in 220 µl phosphate buffer (pH 7.0, containing 1% bovine serum albumin). 100 µl of the dilution were assayed against a cortisol standard diluted in phosphate buffer in six steps of 1:3 dilutions ranging from 20 ng/ml to 82.3 pg/ml (intra-assay coefficient of replicate samples was 16.3% and inter-assay coefficient of variation was 20.4 %). All measurements were well above the detection limit of the assay (lowest amount measured was 7 ng/ml). No saliva sample could be obtained for eight pups on day 1 (four offspring each from individually-housed and group-housed mothers), for five pups on day 8 for the baseline (three from individually-housed mothers and two from group-housed mothers) and for eight pups for the cortisol response (five from individually-housed mothers and three from group-housed mothers).

### Statistical analyses

#### Analysis of litter size, pup growth, and sex-ratio

Data were analysed using mixed-effect models in R 2.13.1 [67] with restricted maximum likelihood estimation and gaussian error distribution, unless stated otherwise. To control for common genetic or environmental effects unrelated to the treatment and avoid pseudoreplication, we included mother ID as a random effect in the models and individual ID nested within mother ID whenever there were repeated measures from the same mothers or the same offspring. Residuals of the models were checked visually for distribution and variance homogeneity by using Q–Q plots. Batch was included as a fixed factor but turned out to be significant only for body weight and cortisol levels (pups from the second batch weighed less, they had higher baseline levels of cortisol and a reduced cortisol response) and was therefore removed from all other models.

The effect of treatment during pregnancy on litter size was analysed by a t-test. Weight and size of pups were also analysed with a lme, including age, age^2^ and age^3^ (for offspring weight only) as covariates to model the changing slope of the growth curve. Additionally, treatment and offspring sex were included as fixed effects and offspring ID nested within mother ID as random effects. Offspring sex-ratios were analysed with a generalized linear mixed-effect model (package lme4), using a binomial error distribution and including treatment as a fixed effect and mother ID as a random effect.

Baseline cortisol was analysed for the day after birth in a model including treatment, sex, and batch as fixed factors, and offspring ID nested within mother ID as random factors. For day 8 we analysed the cortisol baseline level as described above and the cortisol response (the level of the second sample divided by the level of the first sample).

#### Behavioural measures

Six behavioural measures (struggling, latency to leave the hand, activity and number of calls emitted by pups during the social separation test, latency to mother, and exploration activity) were analysed using lmes with gaussian error distribution and restricted maximum likelihood estimation. Latency to mother and struggling were log-transformed to normalize the distributions (after adding 1 to all latency values to allow transformation of zeros). For analyses of the latency to leave the hand, separate variances were estimated for each group (using the varIdent function of the package nlme) since the pups from individually housed mothers were far more variable than pups from group-housed mothers. Approach to novel object in the novel environment test was analysed using a generalized linear mixed-effect model with binomial error structure. For all variables, we analyzed treatment, sex, and their interaction as main effects, while mother ID and offspring ID nested within mother ID were random effects. Data are shown as mean ± SEM, and a p < 0.05 was regarded as significant.

## Declarations

The authors declare that there are no competing interests. Publication costs for this article were funded by the German Research Foundation (FOR 1232) and the Open Access Publication Fund of Bielefeld and Muenster University.

## Authors’ contributions

NvE participated in the conception and design of the study, was involved in the collection of morphological, behavioural data and saliva samples, setup of hormonal and microsatellite analysis, statistical analysis and writing of the manuscript. GJK designed some of the behavioural tests, was involved in the data and sample collection, and contributed to the data analysis, interpretation and literature research for the manuscript. AG took part in the conception and design of the study, the collection of data and samples, performed the statistical analysis and contributed to the writing of the manuscript. All authors read and approved the final manuscript.
